# Enhancing the thermal stability of cowhide jelly through transglutaminase and agar

**DOI:** 10.1016/j.fochx.2026.103946

**Published:** 2026-05-05

**Authors:** Jun Li, Conghui Chen, Peng Wu, Guohong Chen, Xiangren Meng

**Affiliations:** a College of Tourism and Culinary Science, Yangzhou University, Yangzhou 225127, China; b Chinese Cuisine Promotion and Research Base, Yangzhou University, Yangzhou 225127, China; c College of Animal Science and Technology, Yangzhou University, Yangzhou 225127, China

**Keywords:** Gelatin, Transglutaminase, Agar, Covalent cross-linking, Noncovalent interaction

## Abstract

As an important local specialty dish, cowhide jelly suffers from issues such as poor thermal stability. Herein, cowhide jelly was modified using transglutaminase (TG) and agar, and the enhancement effect of the modification on the product's thermal stability and the underlying mechanism were investigated. Results showed that TG and agar significantly improved the melting point (from 29.16 °C to >80 °C), gel strength (from 84.23 g to 116.98 g), and chewiness (from 243.86 mj to 373.97 mj) of cowhide jelly. The covalent forces induced by TG, together with non-covalent forces triggered by agar, increased the cross-linking degree of cowhide jelly to 53.17%. Meanwhile, a large percentage of immobilized water was associated with the denser network structure formed after the cross-linking of TG, agar, and cowhide jelly. Furthermore, secondary structure determination showed that the β-sheet content of cowhide jelly increased (from 33.1% to 40.5%), indicating an improvement in its gelling properties.

## Highlights

• Transglutaminase and agar promoted the melting temperature of cowhide jelly.
• Both covalent and non-covalent crosslinking modes exist in modified cowhide jelly.
• Agar induced non-covalent forces such as ionic bonds and hydrogen bonds.
• Increasing β-sheet content of cowhide jelly improved water-holding capacity.

## Graphical abstract

**Figure:**
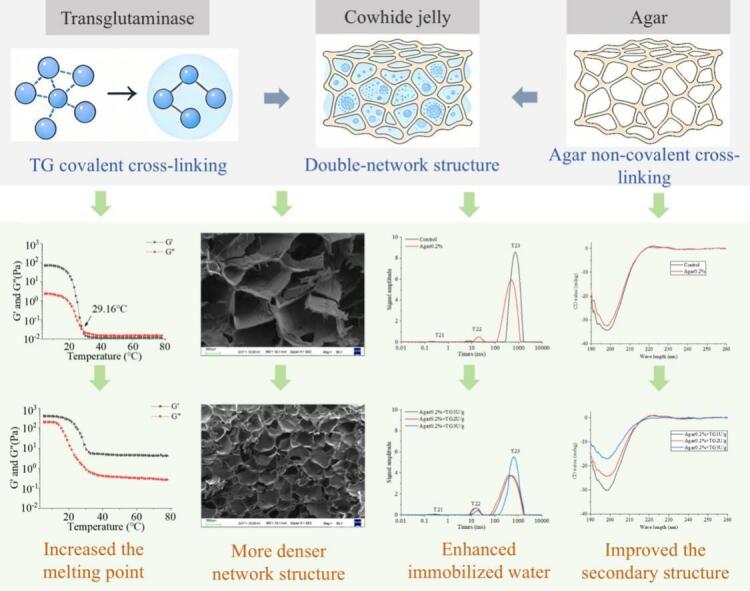

## 1 Introduction

Gelatin is a biodegradable and biocompatible biopolymer obtained by the partial hydrolysis of collagen. Owing to its excellent film-forming, gelling, and emulsifying properties, it has been widely applied in food, pharmaceutical, cosmetic, and biomedical fields (Zahrin, Ishak, Atan, Zainuddin, & Rashid, 2025). As one of the main sources of commercial gelatin, cowhide gelatin accounts for approximately 29% of the global gelatin market and is primarily prepared from by-products of the cattle slaughtering industry (Gao et al., 2022). “Zhenjiang Yao Meat” and “Shuijing Yadong" are specialty dishes of China, which are mainly produced by blending cowhide gelatin with pork, beef, duck, and other ingredients, and are highly popular among consumers (Huang et al., 2014). However, cowhide jelly made from single-source cowhide gelatin exhibited poor thermal stability, which severely compromised product quality and storage stability. The inherent thermal instability of cowhide gelatin stems from the incomplete retention of the native structure of collagen during hydrolysis, resulting in weak intramolecular and intermolecular hydrogen bonds and hydrophobic interactions (Wang et al., 2024). Therefore, improving the thermal stability of cowhide gelatin has become an urgent problem to be addressed in relevant fields.

In recent years, researchers have developed various modification methods to improve the thermal stability of gelatin, including physical modification, chemical modification, and enzymatic modification (Zhang, Li, et al., 2024). Among these methods, enzymatic modification was regarded as a green and efficient modification approach due to its mild reaction conditions, high specificity, and non-toxic by-products. Transglutaminase (TG) is an enzyme that catalyzes acyl transfer reactions, which can form covalent cross-links between glutamine residues and lysine residues of gelatin molecules, thereby improving the structural stability and heat resistance of gelatin (Dai et al., 2024). TG cross-linking significantly increased the gel strength of both high- and low-molecular-weight bovine bone gelatins; in contrast, it decreased the gel strength of gelatins with a higher proportion of α-chains and their aggregates (Gu et al., 2025). Meanwhile, TG effectively promoted the formation of high-molecular-weight components, thereby enhancing the thermal stability of porcine skin gelatin (Du et al., 2021). Besides enzymatic modification, blending gelatin with polysaccharides was also an effective strategy to enhance its thermal stability. Existing studies have demonstrated that TG and carrageenan modification significantly increased the thermal stability and gel strength of fish gelatin by forming the covalent crosslinks (Chen et al., 2022). Moreover, agar, a natural polysaccharide extracted from red algae, possesses excellent thermal stability, gelling ability, and biocompatibility (Beni et al., 2025). It could bind to gelatin molecules through hydrogen bonds and hydrophobic interactions, which contributed to stabilizing the triple-helical structure of gelatin and inhibiting its thermal denaturation (Zhou et al., 2023).

Existing studies have demonstrated that the modification effect combining enzymatic cross-linking and polysaccharide blending was superior to that of single modification methods (Lin et al., 2025). TG modification alone or polysaccharides blending has been proven to improve the thermal stability of gelatin. However, the synergistic mechanism of TG and agar on the thermal stability of cowhide gelatin remained unclear. Therefore, this study aimed to investigate the synergistic enhancement effect of TG and agar on the thermal stability of cowhide gelatin. The influences of TG and agar on the thermal stability of cowhide gelatin were systematically investigated using rheology, texture analysis, interaction force measurement, cross-linking degree determination, sodium dodecyl sulfate-polyacrylamide gel electrophoresis (SDS-PAGE), scanning electron microscopy (SEM), circular dichroism (CD), and low-field nuclear magnetic resonance (LF-NMR). This study was expected to provide a theoretical basis and technical support for the development of high-performance cowhide gelatin products.

## 2 Materials and methods

### 2.1 Materials

Fresh cowhide was purchased from Xiaguang Fresh Food Co., Ltd. (Ningde, China). TG (200 U/g) was obtained from Yuanye Biotechnology Co., Ltd. (Shanghai, China). Agar, Na_2_CO_3_, urea, NaCl, β-mercaptoethanol, 2,4,6-trinitrobenzenesulfonic acid, Tris-HCl, 10% ammonium persulfate, Coomassie Brilliant Blue R-250, 5× loading buffer, tetramethylethylenediamine (TEMED), and sodium dodecyl sulfate (SDS) were purchased from Shanghai Aladdin Biochemical Technology Co., Ltd. (Shanghai, China).

### 2.2 Sample preparation

Washed fresh cowhide was cut into small pieces (1 cm × 1 cm × 1 cm) and soaked in a 2% (*w*/w) Na_2_CO_3_ solution for 30 min to remove excess fat. Then, the treated cowhide was extracted in a water bath at 95 °C for 2 h at a solid-to-liquid ratio of 1:3. The filtered extract was subjected to an oven (Wuxi Maret Technology Co., Ltd., Wuxi, China) at 60 °C for 12 h to obtain cowhide gelatin (the yield of cowhide gelatin extract was 17.94 ± 1.57%, with a protein content of 901.25 ± 41.82 mg/g). The cowhide jelly was prepared by dissolving 4% (*w*/w) cowhide gelatin and other additives in water. The control group only contained 4% (w/w) cowhide gelatin. The agar group added 0.2% (w/w) agar to cowhide jelly. The TG groups added 1 U/g gelatin, 2 U/g gelatin, and 3 U/g gelatin of TG to the cowhide jelly, respectively. The TG-agar groups added 1 U/g TG + 0.2% agar, 2 U/g TG + 0.2% agar, 3 U/g TG + 0.2% agar, respectively.

### 2.3 Gel strength and texture profile analysis (TPA) analysis

The gel strength and TPA were determined by a TA-XT express physical property instrument (Stable Micro Systems Co., Ltd., Surrey, UK) according to the previous studies with minor modifications (Petcharat et al., 2025). All samples were uniformly cut into pieces of 2 cm × 2 cm × 1 cm. The gel strength analysis parameters were as follows: Probe, P/0.5R; decline speed, 1.5 mm/s; test speed, 1.0 mm/s; rise speed, 1.0 mm/s; trigger force, 5 g. The gel strength was defined as the force exerted when the compression depth reached 4 mm. The TPA experiment conditions were as follows: Probe, P50; falling speed, 2.0 mm/s; measuring speed, 1.0 mm/s; rising speed, 1.0 mm/s; compression ratio, 30%; trigger force, 5 g; dwell time in secondary compression, 5 s.

### 2.4 Melting temperature determination

The melting temperature of the samples was determined using an HR-10 rotary rheometer (TA Instruments, New Castle, United States) equipped with a 40 mm diameter parallel plate (Yuan et al., 2023). Briefly, the initial temperature was set at 4 °C, the parallel plate gap was adjusted to 1 mm after the sample solution was loaded, and the system was equilibrated at 4 °C for 20 min. Then, the temperature was increased to 80 °C at a heating rate of 5 °C/min. The strain and frequency were fixed at 1.0% and 0.1 Hz, respectively. The crossover-point of storage modulus (G') and loss modulus (G") during the measurement was considered as the melting temperature.

### 2.5 Intermolecular interactions

First, the samples were incubated at 4 °C for 24 h, and 1.0 g of the sample was minced and mixed with 5 mL of 0.05 M NaCl solution (Sa), 0.6 M NaCl solution (Sb), 0.6 M NaCl +1.5 M urea solution (Sc), 0.6 M NaCl +8 M urea solution (Sd), 0.5 M β-mercaptoethanol (Se), respectively. Then, the samples were mixed thoroughly and incubated at 4 °C for 1 h, and centrifuged at 10000 ×*g* for 15 min. The supernatant was obtained, and the protein concentration was analyzed by the Biuret method. The differences in protein concentration among various sample groups were defined as ionic bonds (Sb-Sa), hydrogen bonds (Sc—Sb), hydrophobic interactions (Sd-Sc), and disulfide bonds (Se-Sd), respectively (Li et al., 2024).

### 2.6 Crosslinking degree

5 mg sample was added into 2 mL 0.1 M NaHCO₃, followed by the addition of 1 mL 0.01% (*v*/v) 2,4,6-trinitrobenzenesulfonic acid. After incubation at 37 °C for 2 h, the reaction was terminated by adding 1 mL 10% sodium dodecyl sulfate and 500 μL of 1 M HCl. The absorbance of the sample was measured at 345 nm using an ultraviolet spectrophotometer (Shanghai Mapuda Instrument Co., Ltd., Shanghai, China), with the group without the sample serving as the blank (Dai et al., 2024). The crosslinking degree was calculated according to the following formula (1):

(1) Crosslinking degree (%) = (1-ODs/ODc) × 100

where ODs denotes the absorbance of agar, TG, and TG-agar groups, and ODc denotes the absorbance of the control group.

### 2.7 SDS-PAGE

Protein samples were adjusted to a concentration of 1 mg/mL, then mixed with 5× loading buffer at a ratio of 4:1. After thorough mixing, the samples were denatured in a water bath at 95 °C for 10 min, followed by cooling and centrifugation at 10,000 r/min for 3 min. The supernatant was collected for loading, with a loading volume of 10 μL.

SDS-PAGE was performed using a 5% stacking gel and a 10% separating gel. Electrophoresis was initially run at 80 V until the proteins entered the separating gel, and then the voltage was increased to 120 V until the bromophenol blue front was approximately 1 cm away from the bottom of the gel. After electrophoresis, the gel was stained with Coomassie Brilliant Blue G-250 and then destained with destaining solution until the background was clear before image capture.

### 2.8 SEM

The gel samples were cut into cuboids of 1 cm × 1 cm × 0.5 cm, then dried in a lyophilizer (Ningbo Scientz Biotechnology Co., Ltd., Ningbo, China) for 48 h, and their smooth cross-sections were obtained with a blade. The samples were fixed on the sample stage with the cross-sections facing upward and then sputter-coated with gold powder. The cross-sections were observed under a SEM (Carl Zeiss, Oberkochen, Germany) with the following parameters: high voltage of 10 kV and magnification of 50× (Chen et al., 2023).

### 2.9 Water distribution

The water distribution of gel samples was determined by a MesoMR23-060H-I LF-NMR (Suzhou Niumag Analytical Instrument Co., Ltd., Suzhou, China) according to the previous study with minor revisions (Bao et al., 2024). Gel samples were cut into 2 cm × 2 cm × 2 cm cubes, quickly placed into NMR-dedicated glass tubes, sealed, and then inserted into the probe coil, ensuring that the samples were closely attached to the tube wall without air bubbles. The Carr-Purcell-Meiboom-Gill pulse sequence was adopted with the following parameters: echo time of 0.4 ms, number of echoes of 16,000, number of scans of 16, magnetic field intensity of 0.5 T. The transverse relaxation time T₂ was measured, and the average value was obtained from three replicate determinations. The proportion of each relaxation peak area to the total peak area was calculated to characterize the relative content of water in different states, thereby evaluating the water distribution characteristics of the gel system.

### 2.10 CD spectrum

Samples were dissolved in distilled water, and the protein concentration was adjusted to 0.2 mg/mL. The CD spectrum scanning was performed by a J-815 spectrometer (JASCO Corporation, Tokyo, Japan) with the wavelength range of 190–260 nm. Distilled water was first scanned as a blank to obtain a baseline spectrum, and the background of the baseline was automatically subtracted during sample scanning. The scanning data were smoothed, and the relative contents of secondary structures were calculated (Wang et al., 2025).

### 2.11 Statistical analysis

All experiments were repeated three times. Statistical analysis was conducted using SPSS 18.0 and Origin 2022 software. The one-way analysis of variance (ANOVA) and Duncan multiple tests were used for significance analysis at a *p* < 0.05 level.

## 3 Results and discussion

### 3.1 Effects of TG and agar on the texture properties of cowhide jelly

The effects of TG and agar addition on the textural properties of cowhide jelly were presented in Table 1. With the increase in TG addition, the gel strength tended to increase, but the change was not significant. Agar significantly enhanced the gel strength of cowhide jelly, and the combined effect of agar and TG was more pronounced. An increase in gel strength indicates a more stable gel network and stronger molecular interactions within the gel system (Chen et al., 2023). The change trend of hardness was consistent with that of gel strength; low TG concentration (1 U/g) increased the hardness of cowhide jelly, while the hardness tended to decrease with further increase in TG concentration. Similar studies also found that the hardness of fish gelatin-TG composite gels decreased with the increase of TG concentration, and the higher the enzyme concentration, the more significant the gel weakening degree (Liu et al., 2025). This might be attributed to the formation of intermolecular or intramolecular covalent bonds catalyzed by TG in gelatin molecules, thereby changing the gel properties of gelatin. However, high concentrations of TG were prone to causing excessive cross-linking (the cross-linking degree rose from 36.15% to 46.63% with the TG content increased from 1 U/g to 3 U/g), hindering molecular aggregation and inhibiting the formation of gel network structure (Fig. 3C-E), ultimately leading to a decrease in hardness (Liu et al., 2020).

**Table 1:** Gel strength and texture properties of cowhide jelly with the addition of TG and agar.

Groups	Gel strength (g)	Hardness (g)	Adhesiveness (g.s)	Springiness (mm)	Chewiness (mj)
Control	84.23 ± 3.39^a^	383.93 ± 8.43^a^	−56.23 ± 2.68^e^	0.90 ± 0.04^b^	243.86 ± 10.14^a^
Agar0.2%	107.80 ± 5.10^b^	569.52 ± 25.65^c^	−86.13 ± 1.90^b^	0.81 ± 0.05^a^	319.00 ± 18.12^c^
TG1U/g	95.93 ± 5.21^a^	427.56 ± 19.51^b^	−66.48 ± 3.04^c^	0.88 ± 0.05^b^	298.81 ± 10.75^bc^
TG2U/g	89.41 ± 1.76^a^	417.58 ± 24.07^ab^	−65.06 ± 1.73^cd^	0.88 ± 0.02^b^	278.04 ± 11.36^b^
TG3U/g	86.18 ± 3.74^a^	412.71 ± 13.93^ab^	−59.55 ± 2.53^de^	0.92 ± 0.02^b^	280.33 ± 14.35^b^
Agar0.2% + TG1U/g	116.98 ± 10.72^b^	723.99 ± 22.41^f^	−98.51 ± 3.02^a^	0.86 ± 0.01^ab^	373.97 ± 13.20^e^
Agar0.2% + TG2U/g	115.63 ± 9.22^b^	684.34 ± 20.47^e^	−102.52 ± 6.08^a^	0.86 ± 0.03^ab^	366.86 ± 8.67^de^
Agar0.2% + TG3U/g	109.74 ± 5.98^b^	639.20 ± 29.81^d^	−100.03 ± 4.11^a^	0.91 ± 0.01^b^	346.10 ± 10.99^d^

Different letters in the same column indicated significant differences (p < 0.05).

Both TG and agar addition reduced the adhesiveness, and the combined addition resulted in a significant reduction in adhesiveness in particular. Agar addition significantly decreased the springiness of cowhide jelly, whereas TG addition exerted no obvious effect on springiness, and the springiness tended to recover when the two compounds were added in combination. Similar studies demonstrated that TG addition not only enhanced the hardness of fish gelatin gels but also preserved favorable springiness (Wu et al., 2025). In addition, the recovery of springiness in the composite gel may also be attributed to the formation of a double gel network. The rigid and brittle first network mediated by agar, together with the soft and ductile second network cross-linked by TG, enables a better balance between elasticity and brittleness of the composite gel (Ahmed et al., 2014). Chewiness reflects the force required for oral mastication of food. The addition of either TG or agar significantly increased the chewiness of cowhide jelly, especially when they were added together.

### 3.2 Effects of TG and agar on the melting temperature of cowhide jelly

**Fig. 1:**
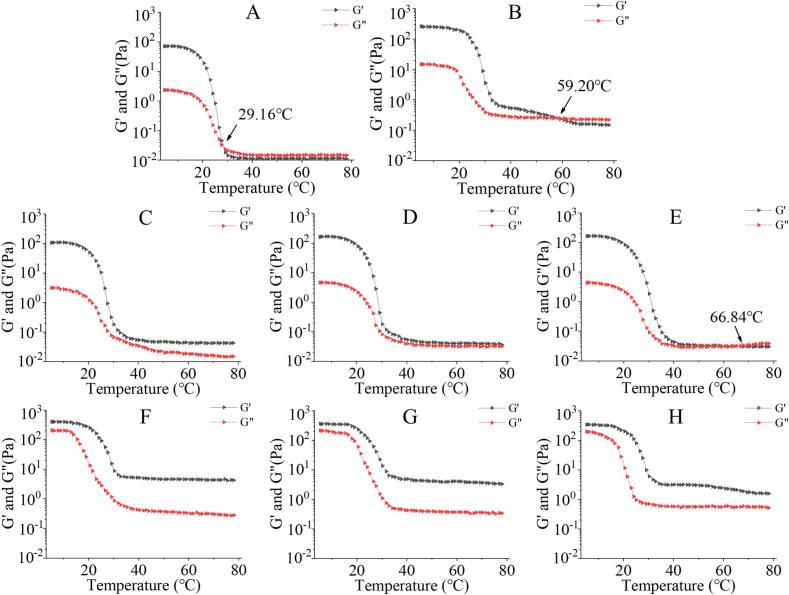
Melting temperature of cowhide jelly with TG and agar addition. A denotes the control group; B denotes the 0.2% agar group; C, D, and E denote the TG groups added 1 U/g, 2 U/g, and 3 U/g TG to cowhide jelly, respectively; F, G, and H denote the TG-agar groups added 1 U/g TG + 0.2% agar, 2 U/g TG + 0.2% agar, 3 U/g TG + 0.2% agar, respectively.

The results of the rheological temperature sweep showed that the melting temperature of the control group was 29.16 °C, at which point the G′ crossed with the G′′, indicating the thermal collapse of the gel network (Fig. 1A). In the group with only agar added (Fig. 1B), the melting point was significantly increased to 59.20 °C. Similar studies found that the affinity of the agar–gelatin complex with water can be enhanced by non-covalent interactions such as hydrogen bonding and electrostatic interactions, thereby improving thermal stability (Zhang, Wang, et al., 2024). When the added amount of TG was 1 U/g and 2 U/g, the G′ and G′′ did not intersect, indicating that TG could impart good thermal stability to gelatin (Fig. 1C and D). This may be attributed to TG catalyzing the formation of ε-(γ-glutamyl) lysine covalent cross-links between protein molecules, and these cross-links are thermally irreversible, thereby enhancing the compactness and thermal resistance of the gel network (Chen et al., 2022). A higher G′ indicates better stiffness or solid-like behavior of the gel, while a higher G′′ implies stronger viscosity or fluid-like behavior. Interestingly, as shown in Fig. 1C-E, G′ exhibited a decreasing trend with increasing TG addition, whereas G′′ showed an increasing trend, implying that the gel showed signs of melting. This observation suggested that the melting temperature of the gel gradually decreased with increasing TG concentration, indicating that excessive cross-linking could compromise the thermal stability of gelatin (Tekle, 2025).

When agar was added in combination with TG, the G′ and G′′ did not intersect, indicating that the composite gel possessed good and irreversible thermal stability (Fig. 1F, G, and H). Meanwhile, both G′ and G′′ were significantly higher than those of the groups with agar or TG alone, suggesting a certain synergistic enhancing effect between agar and TG. The high melting point characteristic of agar dominated the thermal stability of the gel, while the cross-linking effect of TG further optimized the uniformity of the gel network and reduced structural defects at high temperatures (Chen et al., 2022; Voron'ko et al., 2025). In addition, G′ of the complex groups could still maintain a relatively high level in the high-temperature range, indicating that the synergistic effect of covalent cross-linking and the polysaccharide network can delay the thermal melting of the gel.

### 3.3 Effects of TG and agar on the intermolecular forces of cowhide jelly

**Fig. 2:**
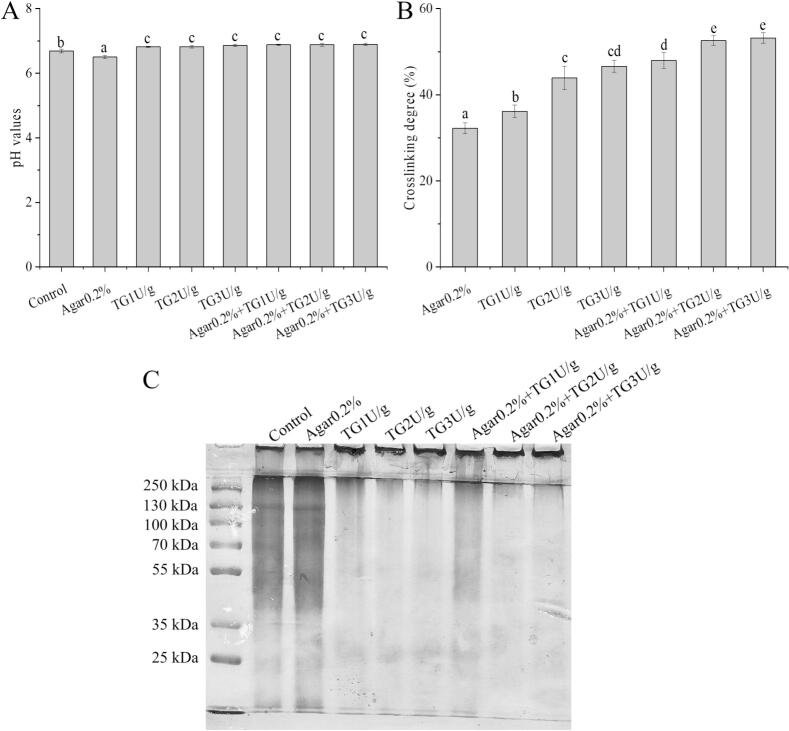
The pH values (A), crosslinking degree (B), and SDS-PAGE (C) of cowhide jelly with TG and agar addition. Different letters in the figure implied significant differences (*p* < 0.05).

This study analyzed the effects of TG and agar on the intermolecular forces in cowhide jelly (Table 2). The results showed that different treatments significantly regulated the proportions of ionic bonds, hydrogen bonds, hydrophobic interactions, and disulfide bonds (*p* < 0.05). In the control group, disulfide bonds accounted for the highest proportion (44.44%), indicating that disulfide bonds were the fundamental intermolecular forces maintaining the gel network of cowhide jelly. After the addition of 0.2% agar, the proportion of ionic bonds and hydrogen bonds significantly increased to 53.57% and 23.15%, respectively, while that of disulfide bonds decreased to 14.68%. This phenomenon may be attributed to the negatively charged groups introduced by the agar, which enhance the electrostatic interactions between protein molecules, thereby increasing the contribution ratio of ionic bonds (Peng et al., 2025). Moreover, the addition of agar reduced the pH of the gel from 6.67 to 6.50 (Fig. 2A), resulting in an increase in the content of cationic ionic bonds and hydrogen bond donors, which might be attributed to the sulfate and carboxyl groups in agar. After the addition of TG, the proportion of disulfide bonds increased from 55.03% to 73.54% with increasing enzyme concentration (from 1 U/g to 3 U/g), while the proportions of ionic bonds and hydrogen bonds decreased significantly (p < 0.05). This phenomenon is consistent with the findings of Wang et al. (2023), who reported that TG-catalyzed cross-linking between glutamine and lysine residues promotes the formation of stable covalent bonds among protein molecules, thereby reducing the dependence on non-covalent interactions.

**Table 2:** The intermolecular forces in cowhide jelly with the addition of TG and agar.

Groups	Ionic bonds (%)	Hydrogen bonds (%)	Hydrophobic interactions (%)	Disulfide bonds (%)
Control	33.07 ± 2.29^c^	17.86 ± 1.98^d^	4.63 ± 0.41^a^	44.44 ± 3.97^d^
Agar0.2%	53.57 ± 1.97^e^	23.15 ± 2.25^ef^	8.60 ± 0.70^e^	14.68 ± 0.86^a^
TG1U/g	21.83 ± 1.98^b^	16.53 ± 1.15^cd^	6.61 ± 0.53^bc^	55.03 ± 2.01^e^
TG2U/g	12.57 ± 1.26^a^	11.90 ± 0.87^b^	5.95 ± 0.38^b^	69.58 ± 2.49^f^
TG3U/g	10.58 ± 0.82^a^	9.26 ± 0.78^a^	6.61 ± 0.71^bc^	73.54 ± 1.36^g^
Agar0.2% + TG1U/g	39.68 ± 3.44^d^	25.13 ± 1.05^f^	7.28 ± 0.24^cd^	27.91 ± 1.43^b^
Agar0.2% + TG2U/g	33.73 ± 3.97^c^	22.49 ± 1.76^e^	8.58 ± 0.72^e^	35.19 ± 3.03^c^
Agar0.2% + TG3U/g	23.15 ± 1.14^b^	15.21 ± 1.03^c^	7.94 ± 0.50^de^	52.62 ± 2.16^e^

Different letters in the same column indicated significant differences (p < 0.05).

When agar was added in combination with TG, the proportion of ionic bonds first increased and then decreased, reaching 39.68% at a TG concentration of 1 U/g, and the pH value returned to nearly neutral at this point (Fig. 2A). This may be attributed to the synergistic effect between the steric hindrance of the polysaccharide and the enzyme-catalyzed cross-linking, which altered the aggregation behavior of the protein molecules (Lin et al., 2025). Hydrophobic interactions accounted for 4.63%–8.60% in all treatment groups, and their proportion in the composite treatment groups was significantly higher than that in the single-treatment groups. This could be attributed to the synergistic effect of polysaccharides and enzymes, which exposed more hydrophobic groups of the protein and enhanced intermolecular hydrophobic association (Fu et al., 2025). Moreover, this study found that when high-concentration TG (3 U/g) was combined with agar, the proportion of disulfide bonds rebounded to 52.62%, suggesting that excessive enzymatic cross-linking may disrupt the electrostatic binding between polysaccharides and proteins, prompting protein molecules to reassociate through disulfide bonds.

### 3.4 Effects of TG and agar on the crosslinking degree of cowhide jelly

This study systematically investigated the effect of combined treatment with TG and agar on the crosslinking degree of cowhide jelly (Fig. 2B). The results showed that the 0.2% agar group exhibited the lowest crosslinking degree (32.23%), whereas the crosslinking degree of samples with TG added alone increased in a graded manner with increasing enzyme dosage (TG 1 U/g, 36.15%; TG 2 U/g, 43.91%; TG 3 U/g, 46.63%). These findings indicate that TG could significantly enhance the stability of the gel network by catalyzing the crosslinking reaction among protein molecules. When agar was combined with TG, the crosslinking degree was further enhanced. Among these combinations, both the agar 0.2% + TG 2 U/g and agar 0.2% + TG 3 U/g groups reached a crosslinking degree of 52.57% and 53.17%, which was significantly higher than that of the individual treatment groups (*p* < 0.05). This synergistic effect is consistent with the findings of Li et al. (2019), who reported that the combined action of TG and polysaccharides can construct a more compact three-dimensional network through the synergy of hydrogen bonds, hydrophobic interactions, and covalent cross-linking.

Additionally, the crosslinking degree of the agar 0.2% + TG 1 U/g group (47.94%) was already close to that of the group treated with a high dose of TG alone (46.63%), suggesting that the incorporation of agar can reduce the required amount of TG. This finding provides a new approach for reducing production costs in industrial applications. Similar studies confirmed that the filling effect of polysaccharides could enhance the efficiency of enzymatic cross-linking, thereby constructing a stable gel network (Ma et al., 2022). Interestingly, when the dosage of TG was increased from 2 U/g to 3 U/g, there was no significant difference in the crosslinking degree of the composite treatment groups, indicating that the crosslinking reaction had reached saturation.

### 3.5 Effects of TG and agar on the protein polymerization of cowhide jelly

The protein polymerization was further evaluated by SDS-PAGE. The protein cross-linking could be reflected by changes in the intensity of protein monomer bands or the protein retained in the wells of the stacking gel (Xu et al., 2022). The bands in the stacking gel wells (Fig. 2C) represented large molecular weight substances that failed to enter the stacking gel. In the control group, the large molecular weight bands were distributed in the range of 100–130 kDa, and the addition of agar did not significantly affect the band profiles. However, after TG addition, most of the bands accumulated in the sample wells and could not enter the stacking gel. This phenomenon was also confirmed by Chen et al. (2023), who found that the covalent cross-linking induced by TG caused protein polymerization, resulting in darker staining in the stacking gel wells.

Therefore, the SDS-PAGE results further verified the covalent cross-linking effect of TG during the gel formation of cowhide jelly, whereas no covalent cross-linking occurred with agar.

### 3.6 Effects of TG and agar on the microstructure of cowhide jelly

**Fig. 3:**
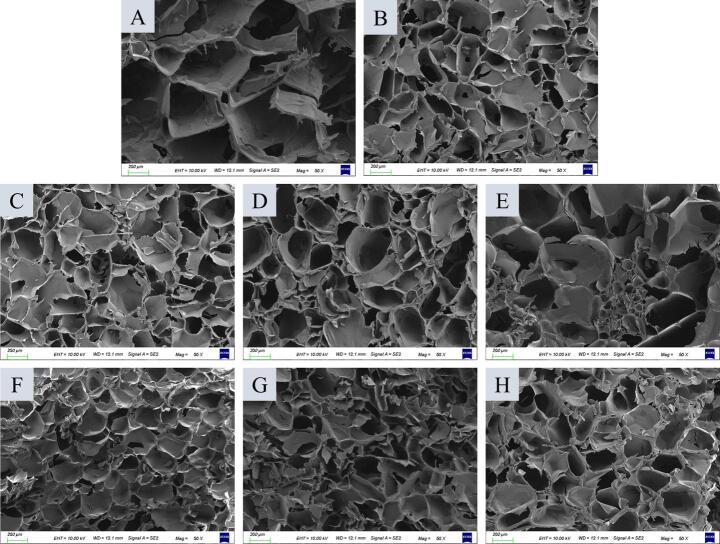
Microstructure of cowhide jelly with TG and agar addition. A denotes the control group; B denotes the 0.2% agar group; C, D, and E denote the TG groups added 1 U/g, 2 U/g, and 3 U/g TG to cowhide jelly, respectively; F, G, and H denote the TG-agar groups added 1 U/g TG + 0.2% agar, 2 U/g TG + 0.2% agar, 3 U/g TG + 0.2% agar, respectively. The magnification was 50 × .

From the SEM images, it can be observed that the internal structure of the control group (Fig. 3A) exhibited large and loose pores, with a sparse and uneven protein network. This indicated that in the absence of exogenous gelling agents, the network structure formed by protein molecules solely through hydrophobic interactions and hydrogen bonds has relatively poor stability. After the addition of 0.2% agar (Fig. 3B), the pores in the sample became significantly smaller and more densely distributed. The agar molecules interweave with the protein chains to form a double-network structure, which remarkably enhances the compactness of the gel. Polysaccharides promoted the formation of protein-protein crosslinking, and the hydrophilic regions of polysaccharide molecules interact with water, subsequently entering the gel matrix as a filler, thereby improving the compactness of the gel structure (Sompongse et al., 2024). As the addition of TG increased from 1 U/g to 3 U/g (Fig. 3C-E), the gel pores first decreased and then increased. This was because TG catalyzed the formation of covalent crosslinks between protein molecules; at enzyme levels of 1–2 U/g, the crosslinking degree is moderate. However, an excessive amount of TG (3 U/g) led to protein over-crosslinking and aggregation, which in turn disrupts the uniformity of the gel network. Excessive enzymatic hydrolysis may no longer be conducive to the formation of a dense gel network structure, and smaller peptide fragments may also cause interactions between exposed active groups, leading to re-aggregation and an increase in particle size (Zheng et al., 2023).

In the composite groups with both 0.2% agar and TG added (Fig. 3F-H), 0.2% agar +1 U/g TG group exhibited the smallest and most uniformly distributed pores. This indicated that the physical gelation of agar and the chemical crosslinking of TG exerted a synergistic effect, which further improved the stability and homogeneity of the gel network. The effect of TG and agar on the gel network structure may be related to the formation of covalent and non-covalent crosslinks between protein molecules and the extensive generation of intermolecular disulfide bonds (Cui et al., 2020; Wang et al., 2023; Zhang et al., 2025). Overall, the combined addition of 0.2% agar and 1 U/g TG was the optimal strategy for constructing a dense and uniform cowhide jelly, providing a theoretical basis for improving the quality of protein gels.

### 3.7 Effects of TG and agar on the water distribution of cowhide jelly

**Fig. 4:**
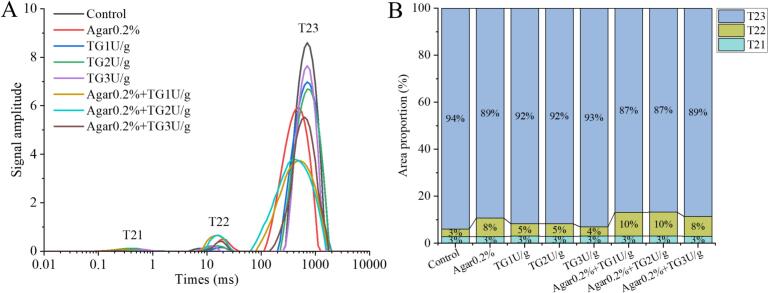
LF-NMR spectra (A) and peak area proportion (B) of cowhide jelly with TG and agar addition.

The LF-NMR T₂ relaxation spectra and water distribution ratio results showed that there were significant differences in water status and migration characteristics among the different treatment groups (Fig. 4). As shown in Fig. 4A, all samples exhibited three characteristic peaks, namely T₂₁ (0.1–10 ms), T₂₂ (10–100 ms), and T₂₃ (100–1000 ms), corresponding to bound water, immobilized water, and free water, respectively (Liu et al., 2025). The control group had the highest T₂₃ peak signal intensity, indicating the richest free water content; whereas the samples with added TG and agar showed a significant decrease in T₂₃ peak height and an increase in the T₂₂ peak area ratio. These results suggested that the protein crosslinking catalyzed by TG and agar could bind more free water and promote its conversion to immobilized water. According to the water distribution proportions shown in Fig. 4B, the free water content of the control group reached 94%, with only 3% being bound water. After adding 0.2% agar, the free water proportion decreased to 89%, while the proportion of immobilized water increased to 8%. This is closely related to the strong water-holding capacity of the polysaccharide molecular network formed by agar (Du et al., 2022).

When TG was combined with agar, such as in the agar 0.2% + TG 2 U/g group, the proportion of free water further decreased to 87%, while the proportion of immobilized water increased to 10%, indicating a synergistic effect between the two additives. Similar studies have found that the combination of TG and gum Arabic increased the water-holding capacity of duck myofibrillar proteins by 8.02%, which might be attributed to the fact that these two additives could promote the aggregation of myofibrillar proteins (Wei et al., 2025). Moreover, when TG and agar were added together, the relaxation time of the T₂₃ peak shifted to earlier values, reflecting a decrease in the mobility of free water and confirming the water-restricting effect of crosslinking (Zhu et al., 2022). Notably, the combined treatment of TG and agar did not significantly change the proportion of bound water, but mainly delayed the loss of free water by constructing a dense gel network.

### 3.8 Effects of TG and agar on the secondary structure of cowhide jelly

**Fig. 5:**
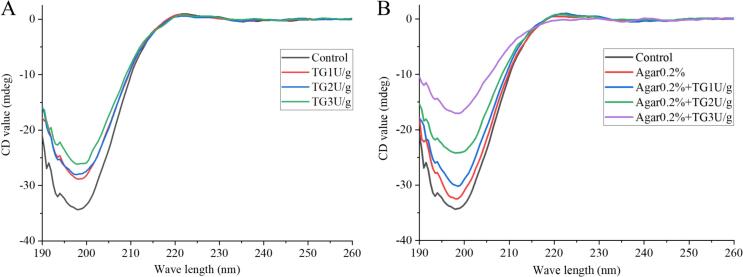
CD spectra of cowhide jelly with TG and agar addition.

The CD spectra (Fig. 5A) showed that all cowhide jelly samples exhibited a prominent negative absorption peak in the range of 190–200 nm, which is a typical characteristic of the β-sheet structure of proteins and consistent with reported secondary structures of gelatin-based materials (Heidary & Soltanizadeh, 2023). Compared with the control group, the samples supplemented with TG or agar showed obvious changes in the intensity of the negative CD signal, indicating that the treatment methods significantly affected the proportions of protein secondary structures (Table 3). When TG was added alone, the α-helix and β-sheet content increased, and the random coil content decreased. When 0.2% agar was added alone, the variation trends of α-helix, β-sheet, and random coil content were similar to the TG groups. When TG and agar acted synergistically, the α-helix content was further increased from 8.1% to 10.2%, the β-sheet content was increased from 33.1% to 40.5%, and the random coil content decreased from 37.8% to 26.6%, indicating that the two compounds had a synergistic effect in regulating the protein conformation. The gelatin in cowhide jelly may maintain its protein spatial conformation through non-covalent interactions, while the covalent cross-linking induced by the addition of TG disrupts the protein conformation, thereby affecting its secondary structure (Chao et al., 2024). Agar mainly binds to the protein through non-covalent interactions, such as hydrogen bonds, to maintain its spatial conformation.

**Table 3:** The contents of secondary structures of cowhide jelly with or without TG and agar.

Groups	α-Helix (%)	β-Sheet (%)	β-Turn (%)	Random coil (%)
Control	8.1	33.1	22.0	37.8
Agar0.2%	8.9	35.7	23.5	31.9
TG1U/g	8.3	36.8	22.3	32.5
TG2U/g	8.5	38.5	22.6	30.4
TG3U/g	8.8	37.3	22.8	31.2
Agar0.2% + TG1U/g	9.3	39.2	22.9	28.7
Agar0.2% + TG2U/g	9.9	39.8	23.1	27.3
Agar0.2% + TG3U/g	10.2	40.5	22.8	26.5

Additionally, α-helix is maintained by intrachain hydrogen bonds, and an increase in its content generally indicates more ordered structures within protein molecules. The β-sheet content is related to hydrophobic interactions in proteins. A decrease in β-sheet content indicates the exposure of internal hydrophobic sites and strengthened hydrophobicity, whereas an increase in β-sheet content results in a slight enhancement or relative stability of hydrophilicity (Zhang, Wang, Cui, Qiu, Ye et al., 2024). Meanwhile, a previous study on surimi gels showed that less random coil content led to improvement of gel strength (Yu et al., 2017). This phenomenon was also confirmed by the gel strength results in the present study (Table 1). Furthermore, the formation of protein aggregates is related to the intermolecular folding structure, and proteins exhibit better gelling properties and water-holding capacity when the relative content of folded structures in their secondary structure is high (Ouyang et al., 2021). Therefore, changes in the secondary structure of the gel, such as increased contents of α-helix and β-sheet as well as decreased random coil content, exerted a positive promoting effect on its thermal stability.

## 4 Conclusion

Overall, the synergistic effect of TG and agar significantly improved the quality of cowhide jelly, which was superior to single modification treatment. The optimal combination was 0.2% agar and 1 U/g TG. Under this condition, the cowhide jelly formed a compact and uniform three-dimensional gel network, with enhanced gel strength and chewiness. The melting point of cowhide jelly exceeded 80 °C, and the thermal stability was greatly improved. The covalent bonds catalyzed by TG, in synergy with non-covalent bonds such as ionic bonds and hydrogen bonds induced by agar, enhanced the cross-linking degree of the complex system, reduced the free water content, and promoted its transformation into bound water. Meanwhile, the addition of TG and agar increased the α-helix and β-sheet contents of cowhide jelly, while reducing the random coil content, thereby improving its gelling properties. Thus, the synergistic modification of cowhide jelly by TG and agar is an effective approach to improving its poor thermal stability. However, further study should focus on the shelf life, sensory quality, and practical application performance of the cowhide jelly, and its potential industrial application value needs to be further explored.

## CRediT authorship contribution statement

**Jun Li:** Writing – original draft, Validation, Methodology, Formal analysis. **Conghui Chen:** Formal analysis, Data curation. **Peng Wu:** Formal analysis, Data curation. **Guohong Chen:** Writing – review & editing. **Xiangren Meng:** Writing – review & editing.

## Declaration of competing interest

The authors declare that they have no known competing financial interests or personal relationships that could have appeared to influence the work reported in this paper.

## Data Availability

Data will be made available on request.
